# ”Daddy comforts me”–Young Swedish children’s perspectives on their family relations before and after their parents’ participation in a parenting programme

**DOI:** 10.1371/journal.pone.0298075

**Published:** 2024-03-15

**Authors:** Anton Dahlberg, Karin Fängström

**Affiliations:** Department of Public Health and Caring Sciences, Uppsala university, Uppsala, Sweden; Karolinska Institutet, SWEDEN

## Abstract

Despite extensive research assessing parenting support, there is a lack of knowledge about the perspectives of the youngest children. In this study, we explored changes in preschool children’s emotional and relational experiences at home before and after their parents participated in a parenting intervention, the Triple P parenting programme. Nine children in total were interviewed, aged 3–6 years, whose parents participated in a group parenting intervention. The interviews were conducted during the first and final group sessions attended by the children’s parents. Data were analysed qualitatively, using a longitudinal approach, resulting in a deductive mapping of the children’s statements onto four themes, based on the parenting intervention’s main objectives. Further, changes in content for each of the four themes were assessed. Before the programme, children described conflicts with siblings, parents’ negative emotions, and punitive parenting behaviours. After the programme, sibling conflicts remained, but parents’ negative emotions decreased and parent threats and violence ceased. Positive family interactions and quality time increased, along with experiences of tenderness and being comforted. Parents also implemented new strategies such as verbal management and more comforting or soothing behaviours. Clinical implications of the results include promoting positive sibling relationships, emphasising parental self-regulation, encouraging empathy and reconciliation, and highlighting the importance of spending quality time with children. These findings contribute to a better understanding of children’s perspectives and provide implications for clinical practice and future research.

## Introduction

Parenting support programmes are common interventions for addressing problems among children and parents. Meta analyses suggest positive effects on a range of behaviours and health-related factors, such as child emotional and behavioural problems; parental stress, depressive symptoms, parenting-related self-efficacy and psychosocial health [1–6]. A common aim among most parental support programmes is to increase positive relationships and behaviours in children and their parents, and to reduce negative interaction patterns and behaviours [7]. Although there is extensive research assessing the programmes from different perspectives and through both qualitative [8, 9] and quantitative [10, 11] methods, there is one important perspective that seldom gets to be explored: the views of the child. Children’s perspectives have been largely overlooked in research, despite their unique insights into their own experiences, opinions and well-being.

There are a number of reasons why it is important to capture children’s perspectives. Children’s descriptions of their experiences, including emotions, can impact the assessment of the child’s well-being and help target interventions [12–15]. In addition, the information children provide on their perspectives can inform practice [12, 14]. But, perhaps more importantly, getting ones voice heard in matters concerning oneself is one of the basic child rights according to the UN *Convention on the rights of the child* [16]. Although there has been increasing recognition of the importance of children’s voices in various research fields, studies involving young children’s views in families seeking parenting support are lacking, leaving a knowledge gap in understanding their perspectives on family relations, and what they stress as important in relation to the content of parenting interventions. In a previous study, the experiences of children whose parents participated in a parenting programme were explored. Children were interviewed prior to their parents’ participation in the programme, and described moments where they and their parents got caught in escalating interaction patterns which resulted in negative parenting behaviours such as yelling and coercion [17]. By involving children in evaluations, we are able to receive opinions and experiences that are not assessable from parents or other adults in the children’s surroundings, as well as getting information that otherwise would not have been obtained.

The aim of the present study was to explore preschool children’s emotional and relational experiences at home before and after their parents participated in a parenting support programme, and how the experiences changed over time.

## Methods

The study was approved by the Regional Ethical board in Uppsala, Sweden (dnr. 2018/188). Findings are reported using the Consolidated Criteria for Reporting Qualitative Research (COREQ) guidelines [18], see S1 File.

### Recruitment and procedure

The participating children in this study were selected based on their parents attending a publicly financed parenting intervention in the municipality of Uppsala, Sweden: the Triple P parenting programme. Triple P is an Australian parenting program based on social learning theory [19]. Triple P aims to enhance parent competence by fostering positive parent-child relationships, encouraging positive behaviours, managing emotional and behavioural issues, decreasing coercive and punitive parenting behaviours, facilitating effective communication about parenting matters, and reducing parental stress [20]. In Sweden, Triple P is offered through brief seminars, individual counselling, and parent groups. For this study, we recruited children aged 3–6 years whose parents attended the parent groups, as this was the only format in Sweden where all programme foci were thoroughly addressed. The Triple P groups consisted of eight sessions, covering aspects such as child development, specific parenting skills and creating personalised parenting action plans. The first four sessions covered specific parenting skills and personalised plans. Sessions 5 to 7 entailed individual phone calls. In the final session, the intervention was summarised and evaluated, motivating parents to sustain acquired skills and changes in parenting behaviours.

The version of Group Triple P in Uppsala is more intense, and parents are actively engaged for a 3.5-to-4-week period. For more details on the demographic make-up of participants during the study period and evaluation of the programme, please see Dahlberg et al. [4]. There were no inclusion or exclusion criteria for attending the intervention, which was free of charge and available in all geographic areas of the municipality. Overall, parents were represented from a variety of social and cultural backgrounds, with participants’ demographics during this study’s time period being representative to the general population. Recruitment took place between November 2018 and October 2019. Written information about the study, covering all important and required aspects of the study according to ethical standards, was sent to parents via email.

Of the 17 children interviewed at the time their parents attended the first group session, nine children were interviewed again at the last group session and they constituted the final sample of the present study. The drop-out of eight children was caused by children getting sick or simply not showing up for the second interview. The study sample consisted of six boys and three girls, median age was 4 years (3 three-year-olds, 4 four-year-olds, 1 five-year-old, and 1 six-year-old).

Parents gave informed verbal and written consent, and all children who participated gave verbal assent. The interviews were conducted in a room adjacent to the room where the parenting sessions took place and only the interviewer and child were present at each interview. All interviews were conducted by either of the two authors, both trained child psychologists and with a thorough experience in child interviewing. The interviewers had no prior relationship to the children. The interviews began with the researcher introducing themselves, obtaining assent, and explaining the interview process. The interviewer established and explained four ground rules: acknowledging the option for the child to express confusion; correcting any inaccuracies on the part of the interviewer; recognising not knowing an answer; and having the choice to end the interview at any time, ensuring voluntary involvement while promoting accurate recall [21]. The language skills among the interviewed children showed significant diversity. Some faced challenges in constructing words and sentences, others demonstrated high language proficiency, while others still had Swedish as their second language. All interviews were conducted in Swedish.

The computer-assisted, visual interviewing aid In My Shoes was used for all interviews together with a semi-structured interview guide. In My Shoes is specifically designed for helping younger children and children with communication difficulties to express themselves and describe their contexts and experiences [22–24]. In My Shoes contains different modules with icons representing people, places, emotions, sensation, speech and thoughts. The modules with visual aids provide a scaffolding and a structure and helps the child to narrate their family, relations therein, and to provide accounts of their own and other people’s behaviour and emotions. The modules can be used flexibly and in the first interview four modules were used with the aim of personalizing and practicing using the method (modules Introduction; Emotions; Emotions and Scenes; People). In order to aid the child to narrate emotional and relational experiences within the family, the Emotions and People module was used (see S2 File for screenshots of the modules used). In the second interview, only this last module was used. Open-ended questions were posed related to both positive and negative emotions experienced in the family and the same questions were asked at the first and second interview (see S3 File for the interview guide). In both interviews the child was prompted to talk about their experiences “here and now”.

The first interviews lasted between 12 to 30 min, with a mean time of 17 min. The second interview length ranged from 6 to 22 min, with a mean time of 14 min. All interviews were videorecorded. The time between the two interviews was approximately 3.5 weeks.

Following Swedish legislature, families where children are suspected to suffer from abuse must be reported to the social services. In this study, the researchers had contact with the social services to consult when a child was deemed to be at risk.

### Data analysis

The interviews were transcribed verbatim by an external transcriber and all names were removed. Data were analysed qualitatively, using deductive, manifest content analysis [25] and building upon a longitudinal approach as outlined by Grossoehme and Lipstein [26] and Saldaña [27]. Grossoehme and Lipstein describe two approaches for analysing longitudinal qualitative data: cross-sectional and trajectory. For this study, a recurrent cross-sectional approach to analysis was applied, with all participants interviewed before and after the parenting intervention. Using an approach in which present experiences were explored at several time points was considered favourable, as it did not require the children to reflect on how something had been in the past, nor to relate it to the present. When conducting a longitudinal qualitative study, change over time is a key concept to include [27]. Change can include both quantitative and qualitative aspects of the phenomenon. In our study we have defined change as the increase or decrease of reported behaviour, experiences and/or emotions in children’s narratives. We have also incorporated qualitative aspects of change, e.g., changes in how children described their experiences, level of detail and what they described.

Four themes were created a priori, based on the main foci of Group Triple P: Negative family relationships, Negative parenting strategies, Positive family relationships, and Positive parenting strategies, constituting a structured matrix. The themes’ titles were supplemented based on what emerged during the analysis, to describe their specific content. The authors systematically read through all interviews condensing manifest content into codes, i.e., units containing the main propositional content. All statements made by the children regarding family experiences were included in the analysis. In line with Saldaña’s longitudinal qualitative analytic approach, the material gathered at timepoint one was compared with the interviews conducted during the initial timepoint [27]. The analysis process was divided into four steps:

1. Individual matrices were created for all children. The matrices were time-ordered and sequential. The codes were mapped to one of the four themes at each timepoint (before and after).
2. Next, all individual matrices were merged into a longitudinal matrix, containing each individual’s codes before and after Group Triple P, across all four themes.
3. Thereafter, the aggregated material was assessed and abstracted to describe the overall content for all themes at each time point.
4. At the final stage, quantitative and qualitative changes in content over time at theme-level were analysed.

#### Positionality statement

Both authors were working as clinical child psychologists and researchers within child health care and parenting during the study period. In addition, the authors have extensive clinical experience and formal training in parenting support programmes, including Triple P. These positions and experiences might contribute to the interpretations of the children’s experiences.

## Results

The four themes Negative family relationships, Negative parenting strategies, Positive family relationships, and Positive parenting strategies are presented below. For each theme the results from the first set of interviews are presented first and the results from the second set of interviews are presented last, together with the quantitative and qualitative aspects of change. Quotes representing children’s descriptions are provided.

### Negative family relationships—Quarrels, anger and absent parents

Before the parents participated in Group Triple P, several children described conflicts with siblings in relation to feeling angry or sad. They explained that their siblings were misbehaving and that this misbehaviour was linked to the conflict situations. Examples of sibling’s misbehaviour was teasing or hitting the child. Children also described their own misbehaviour and how it evoked negative emotions in their parents, such as anger and/or sadness.

*When I get angry*… *everyone is afraid of me* (Boy, 4 years old)

*Mum angry* (Girl, 3 years old)

Children reacted to their parents’ anger with sadness or more anger. Another area related to family relations was children’s descriptions of being alone in the first set of interviews. This included playing alone at home and an under stimulating home environment. One example was a child both describing and showing via In My Shoes that when feeling happy at home he was by himself watching TV.

*At least*, *I like to watch TV* (Boy, 5 years old)

What also emerged was the absence of parents in the children’s narratives, especially the absence of positive time with the parents. Absence was also noticeable in conflict situations where there were descriptions of children isolating themselves or having no parent to comfort them.

After the parents participated in Group Triple P the sibling conflicts still dominated the children’s descriptions of negative family interactions. However, parent’s negative emotions in these conflictual situations were no longer as salient. In this second set of interviews, children’s descriptions of being alone were also less prominent. However, some children gave examples of activities in the family that were not adapted to the child’s level or preferences.

*Not so funny*, *because it was a grown-ups-movie* (Boy, 6 years old)

In the interviews conducted after Group Triple P, children gave more pronounced and nuanced descriptions of their own negative emotions at home. One child explained that things are not so good at home, giving examples of conflicts between the parents.

### Negative parenting strategies—Yelling, punishing and slapping

In the interviews conducted before the parents participated in Group Triple P, children described how their parents responded to their and their siblings’ behaviour with anger. Parents could shout at the them and some children also described parents threatening them and subjecting them to physical violence.

*She says I will be punished* (Boy, 4 years old)

In the interviews conducted after Group Triple P participation, there were no descriptions of either threats or violence. The only physical intervention mentioned was a parent who forcefully carried their child away during a conflict. Despite the absence of physical violence, children still reported that parents were sometimes angry and shouting. Furthermore, children described parents telling them to go to their room (during a conflict), which made them sad and angry. Another parenting behaviour mentioned was allowing the angry or sad child to watch something on the iPad.

*I sit down with the iPad* [when angry with siblings] (Boy, 6 years old)

### Positive family relationships—Cosy time, tenderness and caring siblings

In the first set of interviews, quality time with the parents/family was described by a few of the children. The only concrete example was a child reporting having Friday family cosy time, i.e., eating snacks and watching a movie together. However, some children described positive moments with their siblings and some gave examples of being comforted by them when feeling sad or distressed.

*Because they* [big sister, baby and cat] *are my comforters* (Girl, 4 years old)

One child also mentioned feeling good when he saw his parents having a good time together.

*My mum she- she dances* […] *with my dad* (Boy, 4 years old)

In the interviews conducted after Group Triple P, there were a lot more descriptions of positive family interactions and relations. In particular, quality time was often described and it was a time when the child felt happy. One child expressed a longing for spending more time with his parent. Something that had not emerged in the interviews before Group Triple P was experiences of tenderness. The children described that they experienced tenderness with their parents as well as with their siblings.

*When I go to preschool and when he* [little brother] *comes to me*, *he hugs me* (Boy, 4 years old)

In addition, positive moments between the child and sibling/s, child and parent as well as between parents were also described. These moments made the children feel good and happy. One child also explained feeling happy when spending time with her pets.

### Positive parenting strategies—Keeping calm, comforting and praising

Before Group Triple P, some children reported that parents dealt with unwanted behaviour verbally, i.e., they talked to the child or siblings. One child described that they had house rules at home that told them what not to do, such as hitting their siblings.

*You are not allowed to fight*, *you are not allowed to kick*, *you are not allowed to bite* (Girl, 4 years old)

In a few cases, children were comforted by their parents or by the entire family.

*Then everyone comforts me when I am sad* (Boy, 4 years old)

In the interviews conducted after Group Triple P, children more frequently reported that parents handled misbehaviour and unwanted behaviour verbally. In addition, children gave examples of parents being calm during times of distress, i.e., conflicts or emotional upsetting experiences. More children also reported receiving comfort and reassurance from their parents, and they also gave examples of making up after a conflict.

*Interviewer*: *What happens then* [when feeling grumpy]?

*Child*: *Uh*, *m- daddy comforts me then*. (Girl, 3 years old)

One parental behaviour that emerged in the interviews after parents had participated in Group Triple P was that the parents gave praise to children.

[parents say] *“Well built*!*” That feels good* (Girl, 3 years old)

## Discussion

The aim of the present study was to explore preschool children’s emotional and relational experiences at home before and after their parents participated in a parenting support programme, and how the experiences changed over time. Four themes were created based on the main foci of Group Triple P. The comparison of children’s statements before and after demonstrated some important changes in their family experiences. The most significant change was that children no longer reported threats or violence in the interviews conducted after their parents participated in Triple P. This is in line with the goal of Triple P, i.e., to reduce negative or punitive parenting. Children still experienced, to some extent, their parents being angry and shouting during conflicts. However, the overall impression was that parent’s negative emotional expressions had been somewhat reduced and they managed to stay a little bit calmer. The importance of parents learning how to self-regulate their emotions during conflicts has been stressed in previous literature [18]. However, the fact that this is also highlighted from the children’s own perspectives is both new and of the utmost importance.

According to children’s statements, some parents also appeared to try new strategies to cope with children’s misbehaviour, such as verbal management, time out, or distraction. However, time out was described as appearing to be unplanned and unstructured (parent picking up the child when angry and taking them to their room), and distraction could be questioned as a functional approach. In Triple P, time out is one discipline strategy that, when used effectively, can prevent parental abuse and regulate emotions [28].

Another interesting and important finding was children’s experiences of increased positive family interactions and quality time. This is also a change that can be reflected in what is being taught in Triple P [19, 20]. Some children did report more time together with the family, but that the activities were not tuned in to children’s needs and wishes, which stresses the need for the parent to have the child in mind during mutual activities.

On the positive side, there was the notion that children experienced more tenderness and affection as proactive rather than reactive behaviour from their parents. This could be understood as parents being more active in their relationship building with their children. Children were also more likely to report being comforted by their parents in the second interviews. This is not something that is emphasised within Triple P, but from a psychological perspective, comfort and repair are two very important elements in relationship building and conflict management [29, 30]. Another aspect highlighted by children, but not very visible in Triple P, was conflict with siblings and how it affects both the child and the family. Sibling conflicts seemed to be equally prevalent and continued to cause anger and sadness in the children. Poorly managed sibling conflict is associated with negative outcomes later in life [31, 32], and might therefore be of importance in early parenting interventions.

### Limitations

One limitation is the rather small sample size. Even though 17 children were interviewed at the first time point, only nine of them were interviewed again due to children being ill and/or parents not bringing them to the second interview. The small number of participating children implies that the conclusions of the study should be treated with caution. Further, no data on demographic variables were collected in the study, such as parental education, marital status, or country of origin. Thus, caution is recommended when transferring the results to other contexts.

It is important to note that, even though the interviews were conducted before and after the parents participated in Group Triple P, this is not intended as an evaluation of the effectiveness of the intervention. Rather, children’s experiences have been explored at two time points with the intention to understand more about their perspectives/opinions and changes therein. Worth noting is that the differences in children’s statements have not been described by the children themselves, but are a result of the process of analysis. This was considered the most reliable approach as it is very difficult for young children in general to reflect on change over time and especially on the areas concerned. Although we have reflected the change over time in the parenting program that parents participated in the explanations of change are, as in any other study of change over time, holistic and multifaceted. This implies that there may be other factors influencing the change we have identified. Future studies with repeated interviews over more than two time points, including more children and in families both participating in and not participating in parenting support programmes would be important.

However, even though the results concerning changes in children’s experiences needs to be interpreted with caution, the value and importance of children’s described experiences and emotions should not be underestimated.

In this study, only children were interviewed, which can be seen as a limitation due to the lack of inclusion of other family members. For future studies, an approach that also includes interviews with parents and siblings would be valuable, to capture family dynamics in a more comprehensive way and from several perspectives.

## Conclusions

There was a change over time in children’s descriptions, mainly regarding a decrease in coercive parenting, increases in quality time with parents, and more descriptions of tenderness. This study fills a knowledge gap by assessing the children’s perspectives in relation to family interactions and emotional experiences at home, for children whose parents attend parenting support.

**Fig 1:**
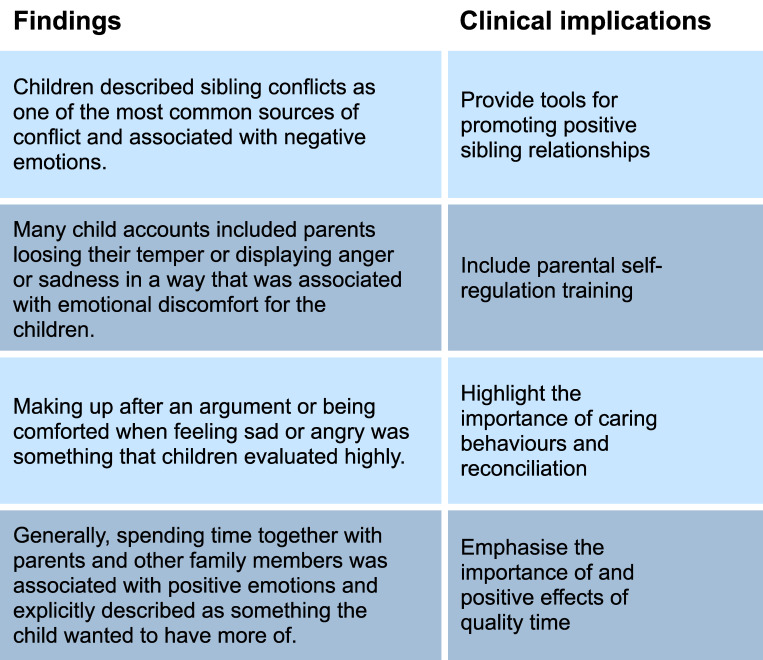
Clinical implications based on children’s experiences.

Based on the findings in this study, we propose four main suggestions for clinical implications of the children’s reports, displayed in Fig 1. Firstly, provide parents with more tools for promoting positive sibling relationships and preventing sibling conflict. This is based on the many descriptions of how the children got into conflict with their siblings and the negative emotions associated with it. Secondly, include parental self-regulation training in the parenting intervention. This, as we saw many accounts of parents losing their temper or otherwise displaying anger or sadness in a way that was associated with emotional distress for the children. Thirdly, emphasise the importance of showing empathy, soothing and caring behaviours, as well as the importance of reconciling after fights or arguments. Lastly, the most prominent reason for children feeling happy when they were at home was spending time together with their parents and siblings. This is a main focus of many parenting programmes, and the results from this study suggest that it is an important factor from a child’s perspective as well.

## Supporting information

S1 File COREQ (COnsolidated criteria for REporting Qualitative research) checklist. (PDF)

S2 File In my shoes screenshots. (PDF)

S3 File Interview guide (English translation). (DOCX)
