# Supplementary material for: ”Daddy comforts me”–Young Swedish children’s perspectives on their family relations before and after their parents’ participation in a parenting programme
Source: PLoS One. 2024 Mar 15;19(3):e0298075. doi: 10.1371/journal.pone.0298075 (PMC10942041; doi:10.1371/journal.pone.0298075)
Supplement: S2 File — (PDF) [file pone.0298075.s002.pdf]

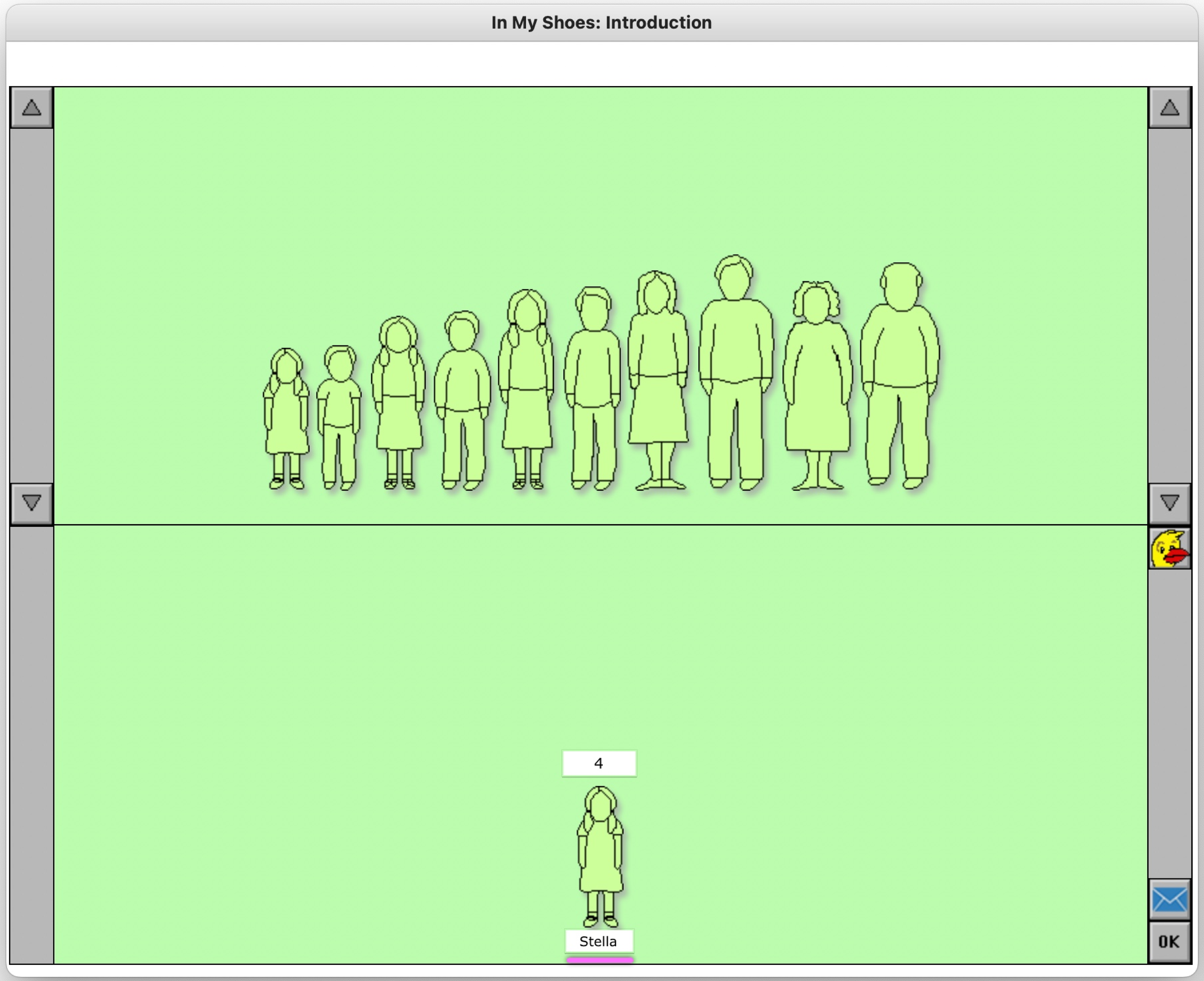
In My Shoes: Introduction
4
Stella
OK

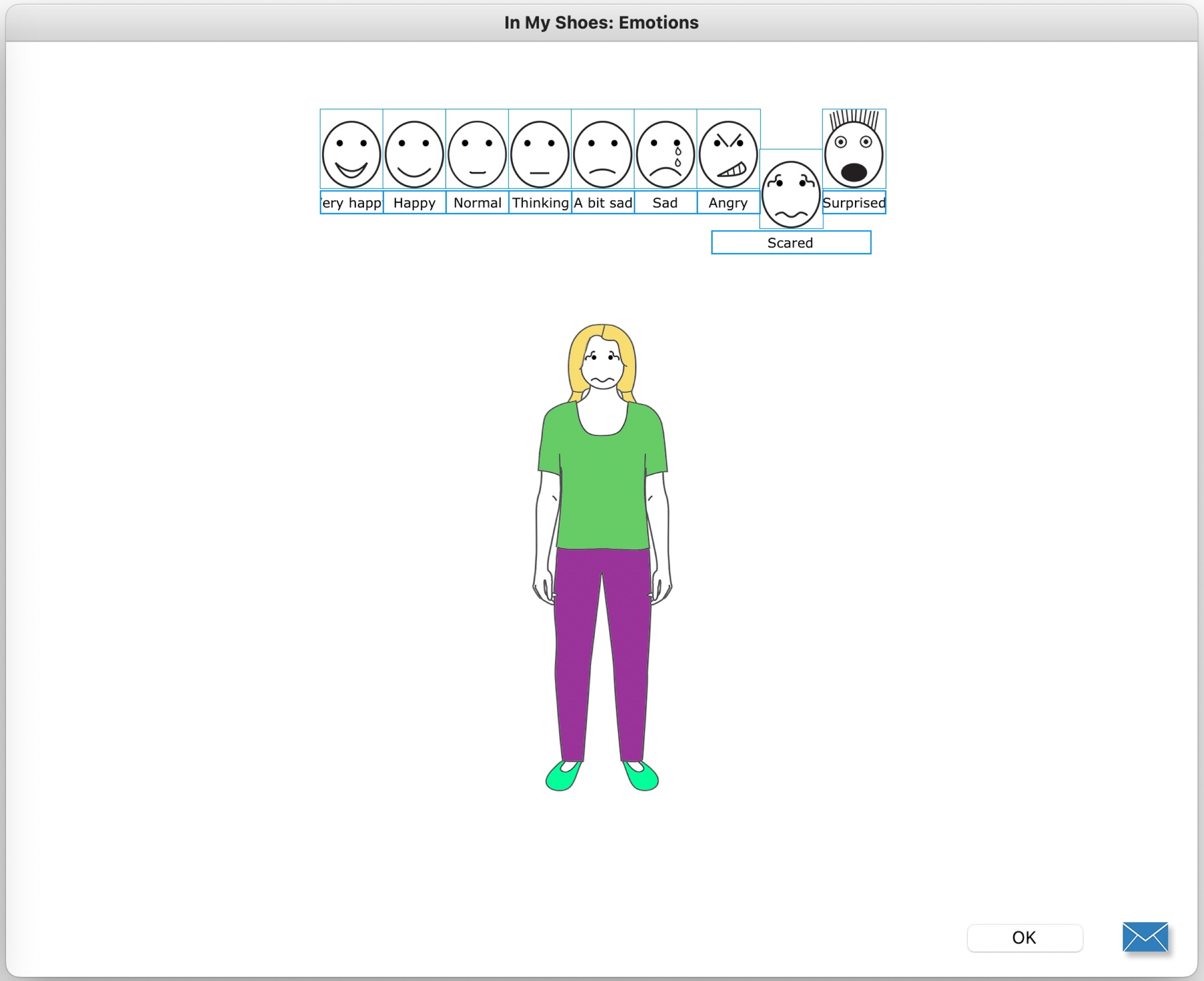
In My Shoes: Emotions
ery happ
Happy
Normal
Thinking
A bit sad
Sad
Angry
Surprised
Scared
OK

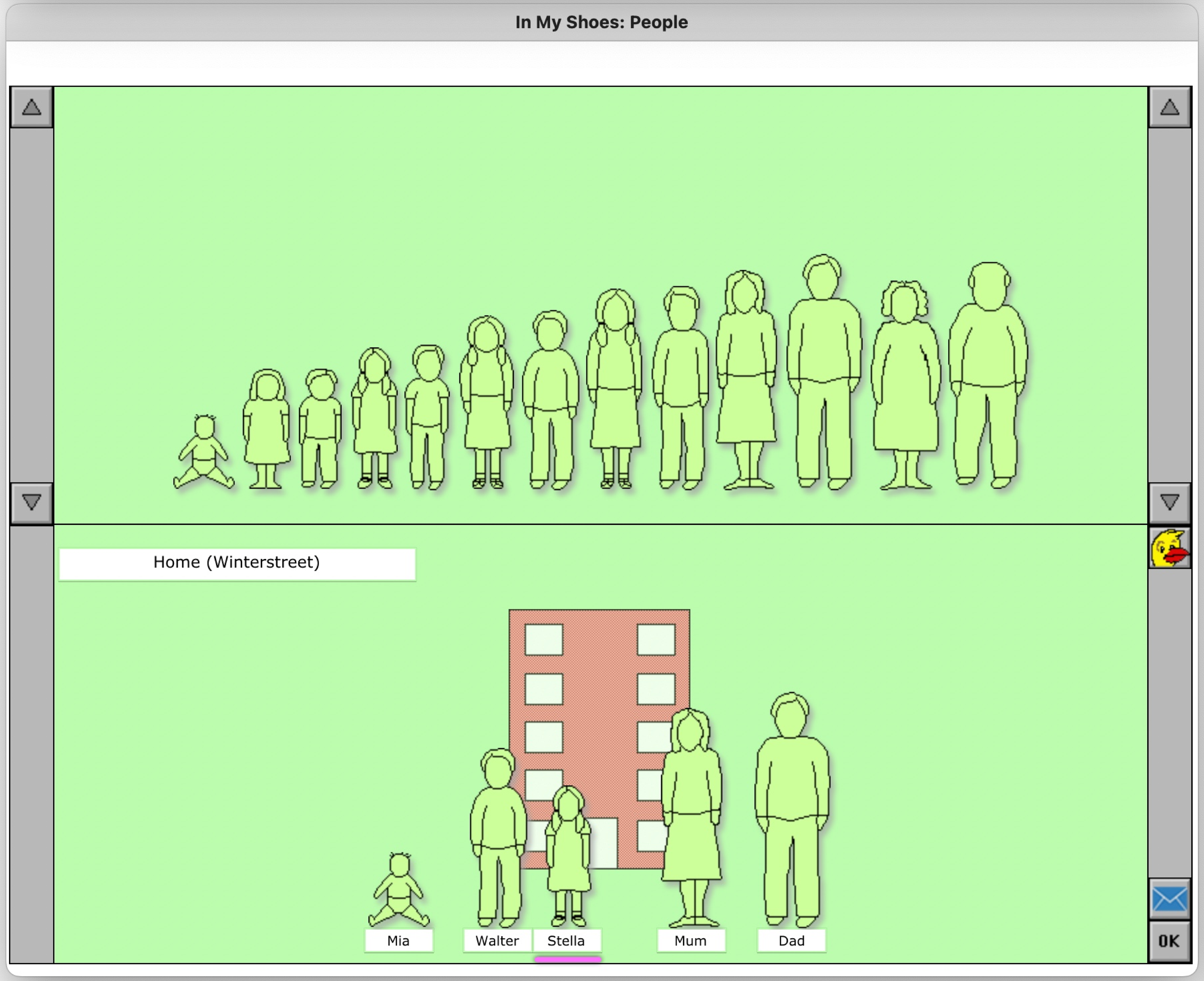
In My Shoes: People
Home (Winterstreet)
Mia
Walter
Stella
Mum
Dad
OK

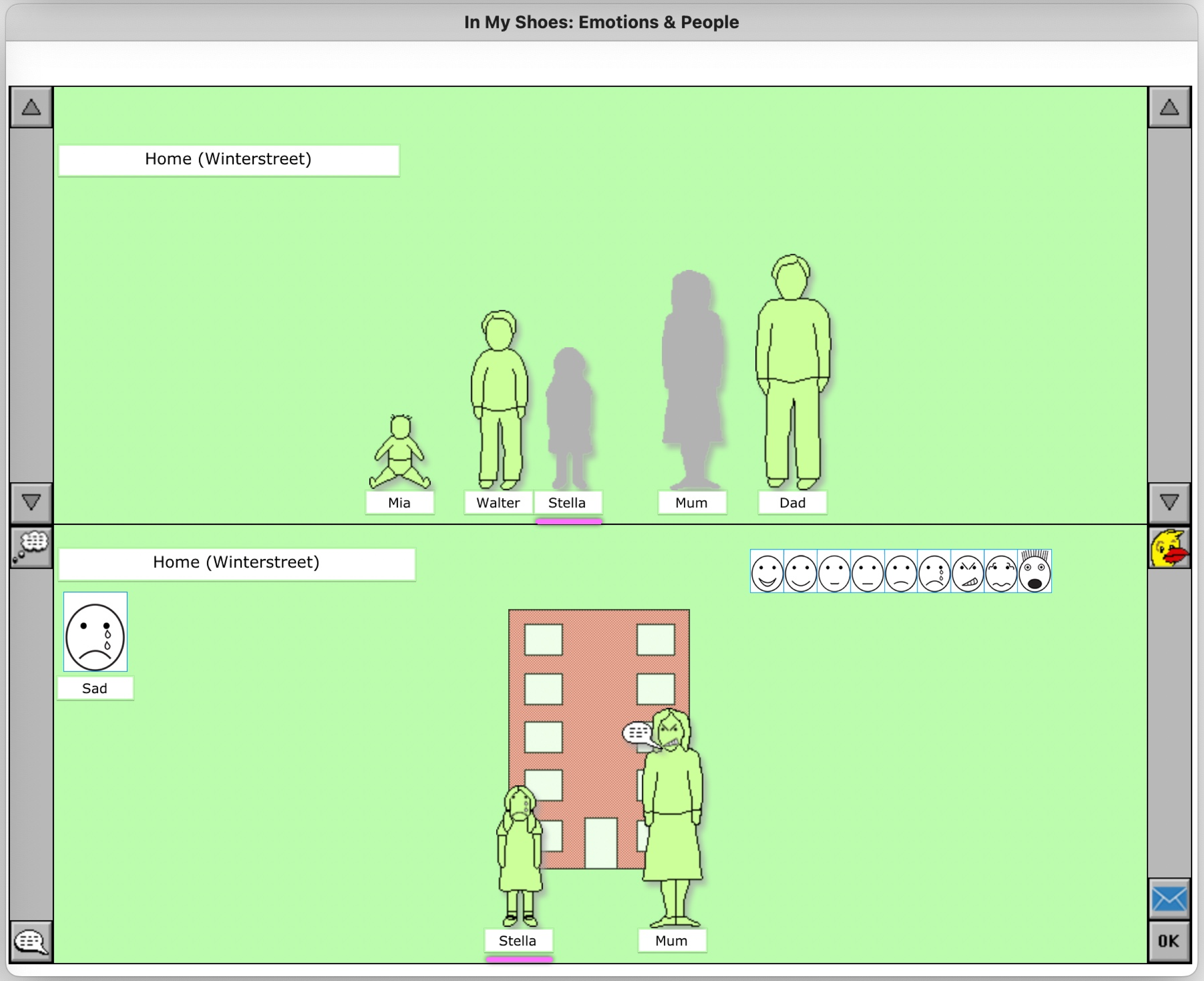
In My Shoes: Emotions & People
Home (Winterstreet)
Mia
Walter
Stella
Mum
Dad
Home (Winterstreet)
Sad
Stella
Mum
OK
