# Supplementary material for: ”Daddy comforts me”–Young Swedish children’s perspectives on their family relations before and after their parents’ participation in a parenting programme
Source: PLoS One. 2024 Mar 15;19(3):e0298075. doi: 10.1371/journal.pone.0298075 (PMC10942041; doi:10.1371/journal.pone.0298075)
Supplement: S3 File — (DOCX) [file pone.0298075.s003.docx]

# Interview guide (English translation)

## Introduction/get to know

Introduce yourself and your role

- My name is…
- I work with talking to children about what they remember and think about different things.

Getting consent - **is it ok for us to talk today?**

Inform about recording - video camera

Rules:

**Don't guess - say don't know**

*If I ask you something that you don’t know the answer to: just tell me. Don’t guess.*

**Say if not understand**

*If I say something that you don’t understand: tell me and I will try to explain better.*

**Tell me if I say something wrong**

*If I say something that is wrong: let me know!*

**If you want to stop, it's ok - say or show it.**

*If you want to stop talking to me: just tell me or give me a signal like this [“stop” sign with hand]*

If your mum or dad wants to know what we have talked about, I can write that down what you tell me on a piece of paper.

## Module 1

Choose one person that looks like you.

## Module 2. Emotions

**Do you recognise any of these?**

**What do you call this feeling?**

**How do you feel inside?**

## Module 3. Emotions and Scenes

Scenes: bicycle, birthday, cat, dog

When the child has chosen an emotion, ask **Why does he/she feel that way? Tell them what has happened.**

Write in the envelope

## Module 6. People

Who lives in your home

## Module 7. Emotions & People

This is when you are at home.

**Do you ever feel this way, that you are [emotion]....?**

**Who is with you?**

**What happened (next)?**

**How did it feel?**

**What did you think then? What do you feel about it?**

**What do you think about it?**

**Tell med more about it.**

Explore all emotions.

## Additional guidelines

### If telling about violence

Adults should not do this to children. Mum/dad needs help to stop.

Is there anything else you want to tell us about at home? Then we're done. Thanks for letting me talk to you. Now I'll give you a tattoo and a cinema ticket to thank you for your help.

### Interview 2, introduction and framing

Now mum and dad and you have been here a few times. Mum and Dad have talked about what it's like to be a parent and learnt things that can make things better for the children and the parents at home. What is it like at home right now?
